# Art Expertise Reduces Influence of Visual Salience on Fixation in Viewing Abstract-Paintings

**DOI:** 10.1371/journal.pone.0117696

**Published:** 2015-02-06

**Authors:** Naoko Koide, Takatomi Kubo, Satoshi Nishida, Tomohiro Shibata, Kazushi Ikeda

**Affiliations:** 1 Graduate School of Information Science, Nara Institute of Science and Technology, Nara, Japan; 2 Center for Information and Neural Networks, National Institute of Information and Communications Technology, Osaka, Japan; 3 Graduate School of Life Science and Systems Engineering, Kyushu Institute of Technology, Fukuoka, Japan; University of Leicester, UNITED KINGDOM

## Abstract

When viewing a painting, artists perceive more information from the painting on the basis of their experience and knowledge than art novices do. This difference can be reflected in eye scan paths during viewing of paintings. Distributions of scan paths of artists are different from those of novices even when the paintings contain no figurative object (i.e. abstract paintings). There are two possible explanations for this difference of scan paths. One is that artists have high sensitivity to high-level features such as textures and composition of colors and therefore their fixations are more driven by such features compared with novices. The other is that fixations of artists are more attracted by salient features than those of novices and the fixations are driven by low-level features. To test these, we measured eye fixations of artists and novices during the free viewing of various abstract paintings and compared the distribution of their fixations for each painting with a topological attentional map that quantifies the conspicuity of low-level features in the painting (i.e. saliency map). We found that the fixation distribution of artists was more distinguishable from the saliency map than that of novices. This difference indicates that fixations of artists are less driven by low-level features than those of novices. Our result suggests that artists may extract visual information from paintings based on high-level features. This ability of artists may be associated with artists’ deep aesthetic appreciation of paintings.

## Introduction

Artists have their own ways of viewing paintings, given their experience and knowledge, which art novices lack [1–8]. To look into this difference, the evaluations of paintings by artists have been compared with that of novices. Evaluations by artists showed a higher correlation than for novices between the quality of artworks and their originality [5]. Artists’ preferences are influenced by expressive qualities such as originality and complexity, while novices are more influenced by emotional features such as pleasantness and warmness [3]. These results suggest that artists attend to different points from those of novices to appreciate paintings. These differences can be thought to be reflected in eye behavior. The eye behavior has been used as a quantifiable indicator of objects or locations toward which visual attention is allocated [9–13].

When viewing representational paintings or photographs, artists tended to fixate more on the background in an image [2, 6]. This result suggests that artists attend to more abstract visual features than semantically informative locations such as a human face and a figure that novices attend to. Artists may have explicit knowledge of non-semantic visual patterns that may not be possessed by novices. From a psychological perspective, compared with novices, artists have superior perception of visual patterns, for example, they can perceive shapes and hidden specific patterns more accurately [14, 15]. However, there is still the possibility that novices may have the knowledge of non-semantic visual patterns. In this case, the semantic objects prevent them from attending to non-semantic visual patterns when viewing representational paintings. In this study, we investigate the existence and role of knowledge of non-semantic visual patterns specific to artists. To do so, we compare visual selection of artists with that of novices during viewing of abstract paintings, since abstract paintings do not include semantic objects. However, to compare their visual selection, we have to quantify whether fixations are attracted to areas related to knowledge of visual patterns or not. The quantification can be addressed by using a computational model based on findings of visual selection in the literature.

Visual selection is implemented by two major mechanisms: stimulus-driven selection and knowledge-based (goal-directed) selection [13]. The stimulus-driven selection is induced by local contrast of visual features such as color or luminance differences [10–12]. The knowledge-based selection is induced by features related to a set of goals in carrying out a certain task [9]. Visual selection is initially stimulus-driven, whereas contribution of knowledge-based factors dominates over time [13]. Knowledge-based factors affect visual selection, because certain visual tasks such as visual search override stimulus-driven factors [16]. Other studies showed good performance of stimulus-driven factors to predict eye fixations in free viewing, implying a high correlation between fixations and local contrast [12, 17].

Based on findings related to stimulus-driven control, computational models to predict fixations have been proposed [18]. One of the most basic models for stimulus-driven control was a saliency map by Itti *et al*. [10, 11]. The saliency map is converted from an input image and based on biological architecture of the early visual system of humans. In the model, according to their proposal, visual attention is assumed to be guided by high contrast locations of three elementary features: color, intensity and orientation [19]. The local contrasts of the three features are represented in a saliency map, which is used as a predictor of fixations in free viewing of an image.

A saliency map, as an indicator of stimulus-driven visual selection, can be used to quantify whether fixations are knowledge-based or not. A saliency map shows expertise affects contribution of stimulus-driven visual selection where fixations of specialist groups showed less correlation to salient areas when they viewed images related to their field [20–22]. This suggests that domain-specific knowledge enhances knowledge-based visual selection, which overrides stimulus-driven factors. Although expertise of artists is less concrete than that of the knowledge in the previous studies and maybe linked with affection or other subjective factors, it is reasonable to assert that artists possess knowledge and expertise that novices do not. Should artists possess such knowledge, stimulus-driven visual selection should have less contribution in artists than in novices. This would then result that their fixations are less attracted to salient areas than those of novices in viewing abstract paintings.

A difference in contribution of stimulus-driven visual selection may be found only in abstract paintings that have few peaks of saliency for the following reason because images with clear saliency tended to have enhanced pop out effect [12]. In our study, according to this result, novices’ fixations should be more attracted to salient areas when viewing paintings with clear saliency than unclear saliency. In contrast, artists’ fixations should be less attracted by saliency independent of the clarity of saliency in the paintings viewed, if their fixations are indeed guided by their knowledge. Therefore, we expect that the difference between artists and novices in term of contribution of stimulus-driven visual selection will be found only in viewing paintings with clear saliency, where the contribution should be lower in artists.

To examine if there is indeed a difference between artists and novices regarding the contribution of stimulus-driven visual selection, we recorded fixation locations during the viewing of abstract paintings and examined consistency between the fixations and a saliency map. This consistency for six artists was compared with that for eight novices. As a metric to quantify the consistency, the receiver operating characteristic (ROC) was used, employing the same method as in [17, 23, 24]. The consistency is referred to as the salience effect in this study. We examined the salience effect in free viewing for five seconds after stimulus onset following the fixation sequence. It is a considerable problem that spatial distribution of saliency in an abstract painting could influence the result [12], and as such the abstract paintings were divided into groups of those with clear saliency and those with inarticulate saliency. For each of the two types of paintings, the salience effects for artists and novices were compared. The result was that the salience effects of the artists were lower than those of the novices when viewing abstract paintings of a few-peaked group.

## Methods

### Participants

Six artists and eight novices participated in our experiments. The artists (three female, *M* = 20.5, *SD* = 0.55) were students of “Kyoto City University of Arts” (one) and “Kyoto Seika University” (five). Their artistic skills were guaranteed by the college entrance examinations they had taken, covering preparatory drawing and sense of color schemes. The artistically untrained participants, novices (four female, *M* = 22.8, *SD* = 1.28), were recruited from non-art departments of “Kyoto Seika University” and “Nara Institute of Science and Technology”: Humanities (two), Biology (three) and Information science (three). The novices were neither especially familiar with drawing nor accustomed to appreciating art works. All participants had normal or corrected-to-normal vision. This study was approved by the Ethics Committee of Nara Institute of Science and Technology. All participants gave written informed consent prior to participation.

### Stimuli

To prevent any influence on eye behavior caused by a bias in painting styles, 20 abstract paintings were collected from various art domains: *Der Blaue Reiter*, *Orphism*, *Cercle et carré*, Futurism, Abstract expressionism, *Art Informel*, *Art Concret*, *Russian Avant-Garde*, COBRA and contemporary art including digital art. No semantic object was depicted in any painting. There were no two paintings by the same artist. Each painting was digitalized and trimmed into a 550×550 pixel square conserving more than half of the area of the whole original image. After this trimming, the paintings were used as stimuli in the experiments.

### Apparatus

For the eye-tracking equipment, we used EyeLink II (SR Research Ltd., Canada), which consists of infrared cameras. The equipment was located in front of a 19-inch monitor, which displayed each stimulus at a screen resolution of 1024×768 pixels. Each participant sat at a distance of 60 cm from the monitor with the head fixed by a chin rest and forehead rest. The visual angle of the stimulus was approximately 22 degrees squared. Vertical and horizontal coordinates of gaze locations of the participant’s dominant eye were recorded at a sampling rate of 500 Hz. After that, the gaze locations were divided into fixations and saccades using a data analysis system where fixation time was set at a minimum of 100 milliseconds and saccade velocity was set at a minimum of 22 degrees per second of visual angle. Stimulus presentation and eye-tracking were controlled using Matlab (Mathworks) toolboxes, psychtoolbox [25] and eyelinktoolbox [26].

### Procedure

Participants were instructed to view freely 20 abstract paintings displayed in turn as the experimental task. They were informed that displayed images were not merely visual patterns but abstract paintings. To distract their attention away from gaze behavior, they were also told that their pupil size would be measured during the experiment. Prior to the recording, calibration was conducted by collecting fixations on target points, where the participant gazed on each of nine points located on 3 × 3 grids on the screen. Drift collection was conducted before displaying each painting so as to maintain measurement accuracy of gaze locations. One of the 20 paintings was displayed in each trial, which consisted of a blank period of one second and a presentation period of 20 seconds. In three previous studies [4, 6, 27] showing eye movements specific to artists when viewing paintings, the viewing periods were set to a long duration of more than 12 seconds. Therefore we set the viewing period to 20 seconds. In the blank period, a white cross was displayed at the center of the screen for one second, and the participants fixated on it. Subsequently, the participants viewed each of the 20 paintings in turn, with gaze locations of the dominant eye recorded. The total number of 20 trials was implemented to show 20 paintings. Data from trials in which recorded gaze locations landed outside of the painting area were excluded from the following analyses.

### Data analysis

#### Saliency map

The saliency map proposed by Itti et al. [10] was used to measure the influence of the stimulus-driven factor in visual selection. The map combines information from three conspicuity maps, each with one of the following features: color, intensity and orientation. To compute each of the three conspicuity maps, the feature values of an input image are used to create nine-scale maps of the feature map with low-pass filters [28]. Then, “center-surround” operations are conducted by calculating decrements between fine and coarse scale maps. After that, the decrement maps are combined into a conspicuity map, which represents the local contrast of the feature in an input image. The three conspicuity maps are finally normalized and summed into a single map, which is the saliency map [10]. In this study, the saliency map was computed by using Matlab toolbox [Harel J. Graph-Based Visual Saliency (MATLAB source code). http://www.vision.caltech.edu/harel/share/gbvs.php].

To combine the three conspicuity maps, the weighting coefficients have to be determined. The weighting coefficients can be set as following two types of procedures: (1) optimize the weights to maximize prediction performance of the map to actual human fixations [29]; (2) set equal weights for the three maps. Each of the weighting coefficients was one third for the saliency map [10]. The equal weight strategy (2) was shown to have poor performance [30]. In the analysis of the salience effects for the first five seconds, the effects were examined for both the saliency map and each of the conspicuity maps. The results of the salience effects in map types defined which procedure we used. We used procedure (1) if map types influenced the result, and procedure (2) if the effects were not considered to be varied in the map types.

#### Quantification of sparseness for saliency maps

Differences in characteristics of the saliency maps across paintings can influence salience effects. As discussed in the Introduction, differences in salience effects in artists compared with those of novices can only be observed in the case of paintings with clear saliency between the peaks and the background. Therefore in this study, salience effects were examined in each of the two groups of paintings (high/ low clarity of saliency). To quantify the clarity, the previous study [12] focused on the difference between mean saliency of each participant, averaged over the viewed paintings at the first fixation, and the mean saliency averaged over pixels in each whole image, again averaged over all paintings used. However, this difference was not able to express the clarity of saliency in peaks against the surround areas. Therefore in this study, the contrast was quantified by calculating sparseness [31]. Sparseness represents the proportion of non-zero values found in a vector. Sparseness can be thought to effectively the clarity of such peaks when, due to the property of a saliency map, the peaks are not distributed too close to each other. In preliminary processing step of computing the saliency map, decrements were calculated between feature maps of different spatial scales, each of which was low-pass filtered by Gaussians ranging from 1:4(scale two) to 1:256(scale eight) [28]. Because of the blurring process, the saliency peaks will not be close to each other. Each pixel of a saliency map contained a salience value *X* which is a scalar value between zero and one. The sparseness of the saliency maps was calculated with *L*_1_ and *L*_2_ norm of the saliency values as in the following equations.

$$sparseness=\frac{1}{\sqrt{N}-1}\left(\sqrt{N}-\frac{L_{1}}{L_{2}}\right),$$ (1)

$$L_{1}=|X_{1}\left|+\right|X_{2}\left|+\right|X_{3}\left|+\cdots +\right|X_{N}|,L_{2}=\sqrt{|X_{1}{|}^{2}+|X_{2}{|}^{2}+|X_{3}{|}^{2}+\cdots +{\left|X_{N}\right|}^{2}}.$$

**Fig 1:**
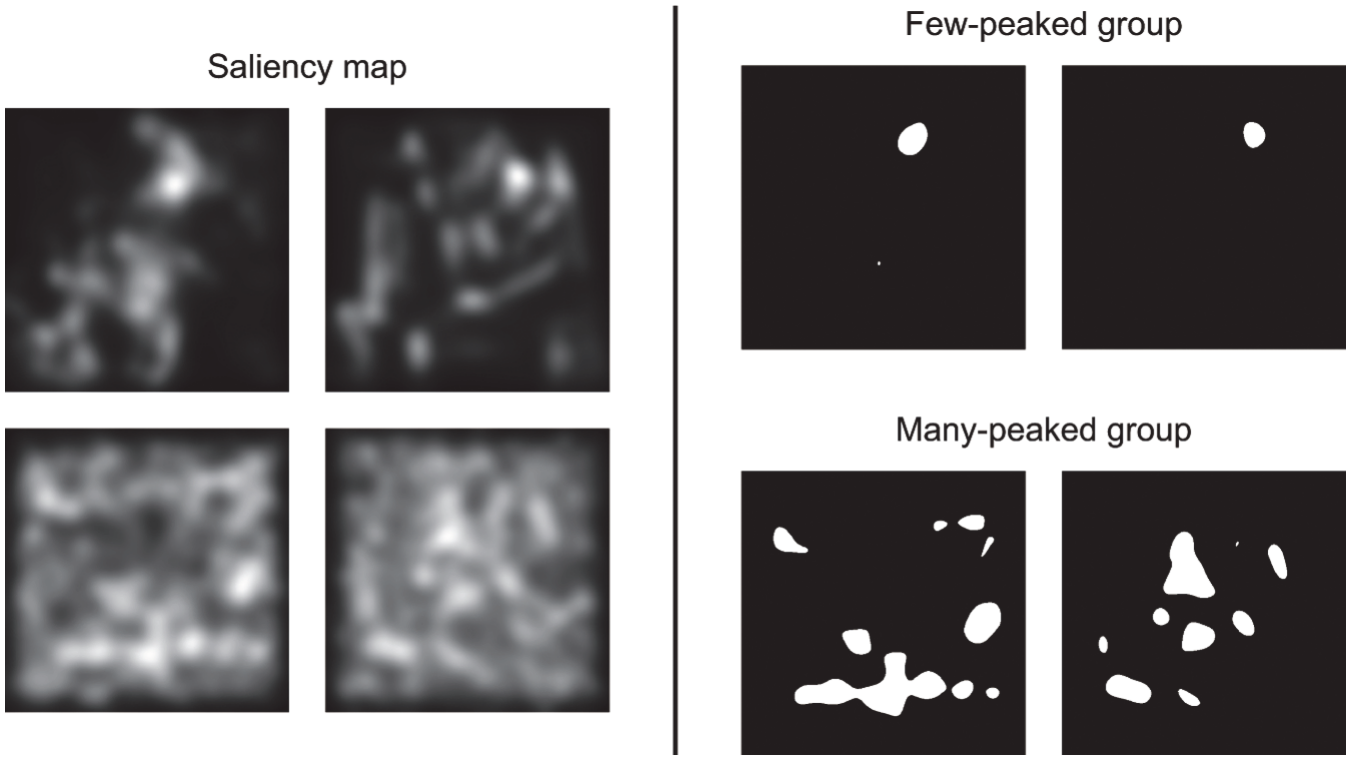
Examples of saliency maps. 20 saliency maps were divided into two groups, a few-peaked group and a many-peaked group, with sparseness of the saliency map.

*N* represents the number of the elements in the vector, which is the number of pixels in a painting here. The sparseness was calculated for each of the 20 paintings. After that, the median of the sparsenesses was used as a threshold to divide the paintings into two groups: paintings exceeding the threshold were classified as having peaks with clear saliency. Images with clear saliency often show to have fewer peaks of saliency over all, compared with those with inarticulate saliency [12]. Therefore we call the 10 paintings with clear saliency a “few-peaked group” and the other 10 paintings a “many-peaked group”. Examples of both groups are shown in Fig. 1. The sparseness can also be calculated for each of the three conspicuity maps, where the *X* is a scalar value of conspicuity at each pixel. Regarding the three conspicuity maps, as an important problem, grouping results could differ among the four maps; the three conspicuity maps and the saliency map. This is due to a difference in the distribution of the sparsenesses for the 20 paintings among these four maps. Actually, the grouping results showed differences among the four maps, though the differences were slight (from zero to two of each group). Therefore in this analysis, we used the grouping results of the saliency map to divide the paintings into two groups.

#### Evaluation method

To quantify the contribution due to stimulus-driven factors in gaze control, we evaluated the predictive performance of a saliency map with that of human eye fixations. Although other evaluation methods have been proposed [18], we used the receiver operating characteristic (ROC) curve, one of the most basic evaluation methods, which had also been used in [17, 23, 24]. The ROC curve was obtained by plotting the true positive rate (*TPR*) versus the false positive rate (*FPR*) having defined the ground truth as the spatial distribution of human fixations and the prediction as a binary saliency map. The equation is as follows.

$$TPR=\frac{N(F_{p}S)}{N(F_{p})},$$ (2)

$$FPR=\frac{N(F_{n}S)}{N(F_{n})},$$ (3)

where *F*_*p*_ denotes the number of fixations and *S* denotes a value of the binary saliency map at each pixel. *F*_*n*_ denotes a value at each pixel which is equal to 1 if the pixel is not fixated, or to 0 if the pixel is fixated. *N*(*X*) takes the summation of *X* over all pixels in a saliency map. The *TPR* and the *FPR* were plotted against one another, while the threshold of the saliency map was varied in the range of saliency values which is from zero to one. Having calculated the ROC curve, the area under the curve (AUC) was obtained. The AUC indicates performance of discrimination between fixated and non-fixated locations by using a saliency map converted into binary using a threshold. With perfect discrimination, the AUC is 1, and when discriminating at chance level, the value is 0.5. This AUC value is called “salience effect” in this study, and calculated for each subject and each painting. The salience effect represents the contribution of the stimulus-driven factor in controlling gaze.

#### Salience effect for the first five seconds

Contribution of stimulus-driven visual selection has been examined in several studies using viewing periods of less than 20 seconds, with the previous studies regarding stimulus-driven visual selection considering a viewing period of five seconds [12, 17]. Doing so ensures that novices’ fixations follow a stimulus-driven factor in that period. Our hypothesis is that the artists’ fixations are guided by a knowledge-based factor while those of novices are stimulus-driven. As such, the viewing period used was selected to allow for analysis of the contribution of stimulus-driven factors. In our analysis, the contribution was initially examined by using three conspicuity maps. The salience effect for the first five seconds of the viewing period was calculated for each participant, and for each painting by using each of these three conspicuity maps. As mentioned above, the saliency map is a single map combining three conspicuity maps. To confirm that the results were not unduly influenced by any of the weighting coefficients of the three maps, a two-way ANOVA was conducted for map types and participant types (artists and novices). When no significant difference in the interaction between map types and participant types was found, the salience effect was also calculated for each participant and each painting by using a saliency map which was combined with the three conspicuity maps equally. The salience effects for the artists were compared with those of the novices. In addition, the salience effects were also compared with those of random fixations. The random fixations were sampled from a Gaussian distribution estimated from the actual fixations for all participants and for all paintings in the first five seconds after onset.

The above procedure was implemented for each of the two painting groups, the few-peaked group and the many-peaked group. Relevant variables are explained in Table 1, Table 2 and Table 3 in the results section.

**Table 1:** 2-way ANOVA of salience effects for few-peaked group.

	Sum Sq.	d.f.	Mean Sq.	F score	Sig.	Partial *η* ^2^
Subject type	0.37	1	0.37	18.97	***	0.04
Map type	1.16	2	0.58	29.36	***	0.12
Interaction	8.48e-04	2	4.24e-04	0.02	NS	8.86e-05
Error	8.01	405	0.02			
Total	9.57	410				

*** and NS indicate significance at p < .001 and no significant difference, respectively.

**Table 2:** 2-way ANOVA of salience effects for many-peaked group.

	Sum Sq.	d.f.	Mean Sq.	F score	Sig.	Partial *η* ^2^
Subject type	0.01	1	0.01	0.61	NS	1.52e-03
Map type	0.09	2	0.05	2.85	NS	1.40e-02
Interaction	0.02	2	0.01	0.70	NS	3.46e-03
Error	6.61	399	0.02			
Total	6.73	404				

NS indicates no significant difference.

**Table 3:** Mean and SD of salience effects for saliency map with equal weights.

		M	SD	Sig.	d (effect size)
Few-peaked group	artists (n = 59)	0.70	0.15	**	0.50
	novices (n = 78)	0.77	0.13		
Many-peaked group	artists (n = 57)	0.70	0.10	NS	0.11
	novices (n = 78)	0.69	0.13		

** and NS indicate significance at p < .01 and no significant difference, respectively.

#### Time variation of salience effects

We also compared artists and novices for the temporal sequences of the salience effects. The sequence consists of the salience effects at each consecutive fixation calculated for each participant and for each painting. The goal of this analysis was to see when there was a difference in salience effects between artists and novices. First, we examined whether a difference in salience effects was shown across the two main elements: participant types and fixations. A two-way ANOVA was conducted across these two effects for each painting group. Then, for each fixation, comparisons of salience effects were made between artists and novices. In comparing artists and novices, however, the temporal distribution of the salience effects is affected by large variance in time. Therefore, every three successive fixations were bundled into one, termed a “bundle”, and each of the bundles was used to make the comparisons.

In these analyses, we used 30 fixations, eliminating the first fixation, in which participants fixated on the white cross. The number of the fixations during the viewing period, 20 seconds, was different across both participants and paintings. For a fair comparison, the number used in the analysis was arranged to maintain the following two conditions: First, over an index of fixations starting at 1 and ending at the largest number of fixations observed for any painting/participant combination, we include in our analysis only the indices for which over 90% of the painting/participant occurrence yielded an observation. This was done independently for artists and novices. Second, the number of indices used must be multiples of three so that every three may be bundled.

## Results

### Salience effect in the first five seconds

**Fig 2:**
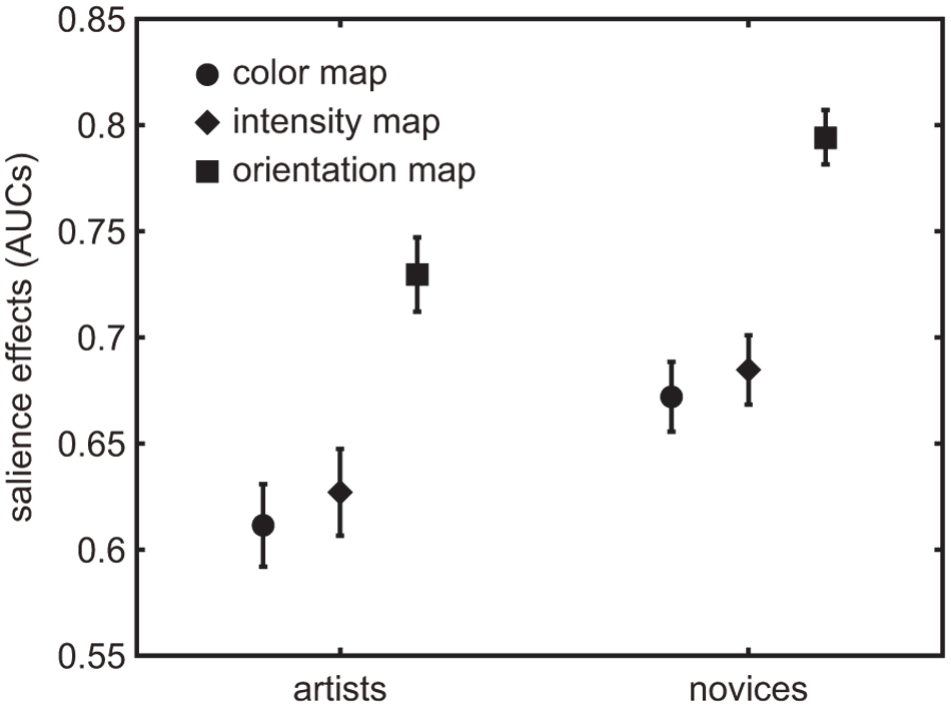
Salience effects of three conspicuity maps in the first five seconds for a few-peaked group. Each symbol represents the mean salience effect of each of three conspicuity maps. The error bars represent standard error.

**Fig 3:**
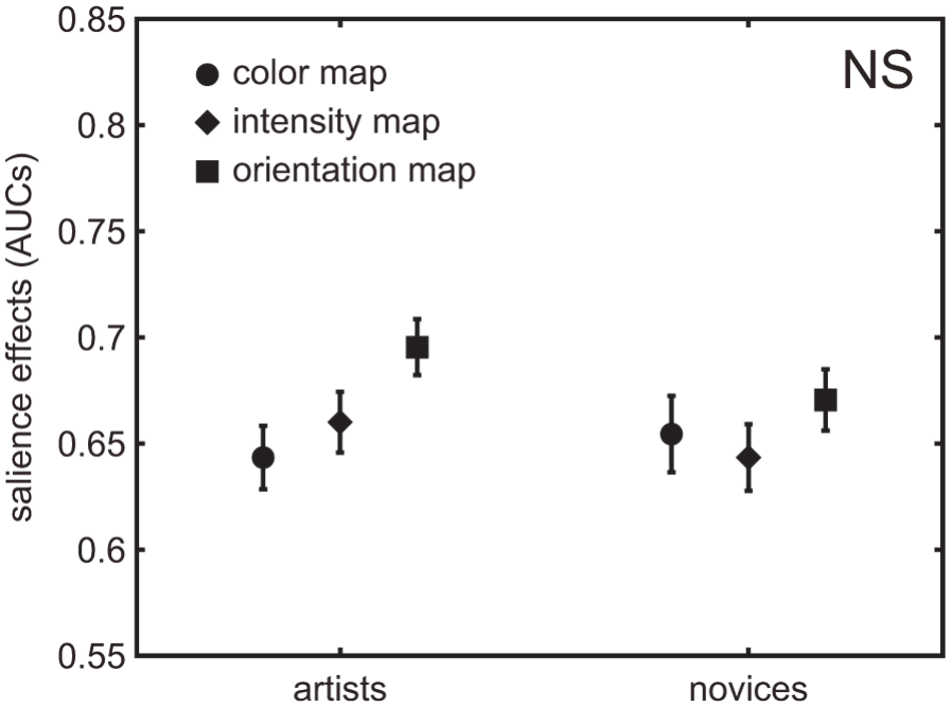
Salience effects of three conspicuity maps in the first five seconds for a many-peaked group. Each symbol represents the mean salience effect of each of three conspicuity maps. The error bars represent standard error. ns means no significant difference was shown.

In the few-peaked group, a two-way ANOVA confirmed a significant difference in salience effects across both of the main factors: map types and participant types (artists and novices). The salience effects of the artists were significantly lower than those of the novices, *F*(1,405) = 18.97, *p* < .001. The salience effects in map types also showed a difference *F*(2,405) = 29.36, *p* < .001, but no significant interaction was found *F*(2,405) = .02, *p* >.05 (Fig. 2). On the other hand, in the many-peaked group, a two-way ANOVA showed no significant differences in either of the two factors nor in their interaction (Fig. 3). As a result, the weighting coefficients of conspicuity maps did not influence the difference between participant types. The results are also shown in Table 1 and Table 2.

**Fig 4:**
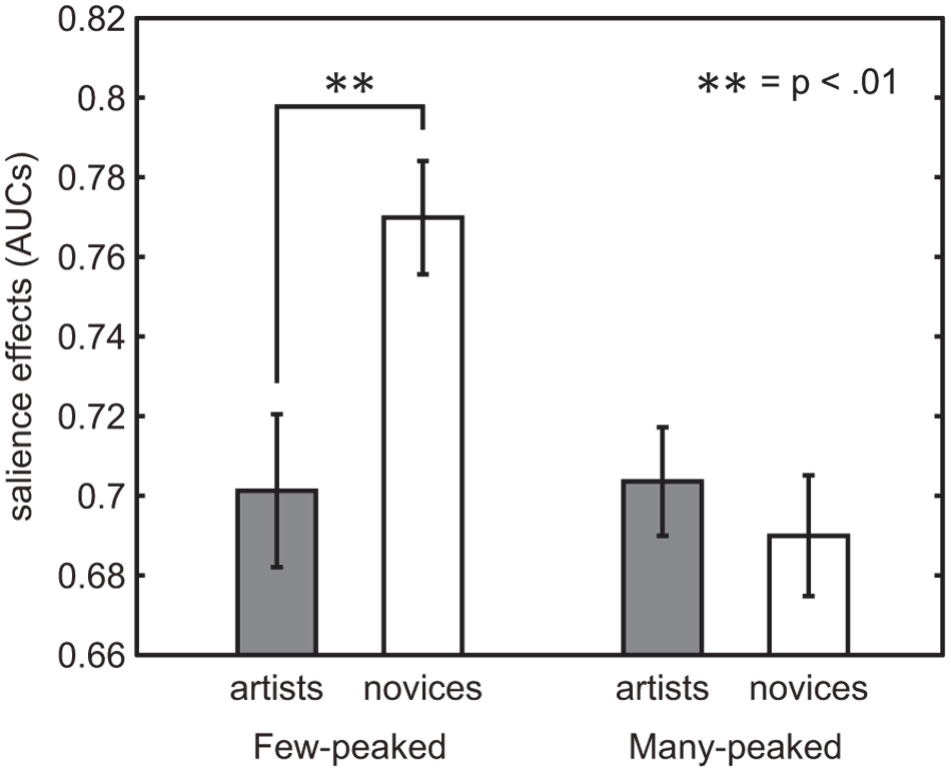
Salience effects of saliency map in the first five seconds for a few-peaked group and a many-peaked group. Each bar represents the mean salience effect of a saliency map. The gray bar is for the artists and the white bar is for the novices. The error bars represent standard error.

Salience effects of saliency maps with uniform weighting coefficients were lower in the artists than in the novices for the few-peaked group, *t* test, *p* < .01, but the difference was not shown in the many-peaked group, *t* test, *p* = .75 (Fig. 4). The results are also shown in Table 3. Compared with random fixation, the salience effects of both the artists and the novices were higher in both the few-peaked and many-peaked groups (*p* < .001).

### Time variation of salience effects

In viewing paintings of the few-peaked group, two-way ANOVA showed a significant difference across participant types, *F*(1,4003) = 36.45, *p* < .001, and in fixations, *F*(29,4003) = 3.13, *p* < .001, but not in the interaction between the two factors, *F*(29,4003) = .93, *p* = .57. The salience effects of the artists were significantly lower than those of the novices. In viewing paintings of the many-peaked group, a significant difference was shown in fixations, *F*(29,3953) = 2.53, *p* < .001, but not in participant types, *F*(1,3953) = 2.11, *p* = .15 and their interaction, *F*(29,3953) = 1.00, *p* = .47.

**Fig 5:**
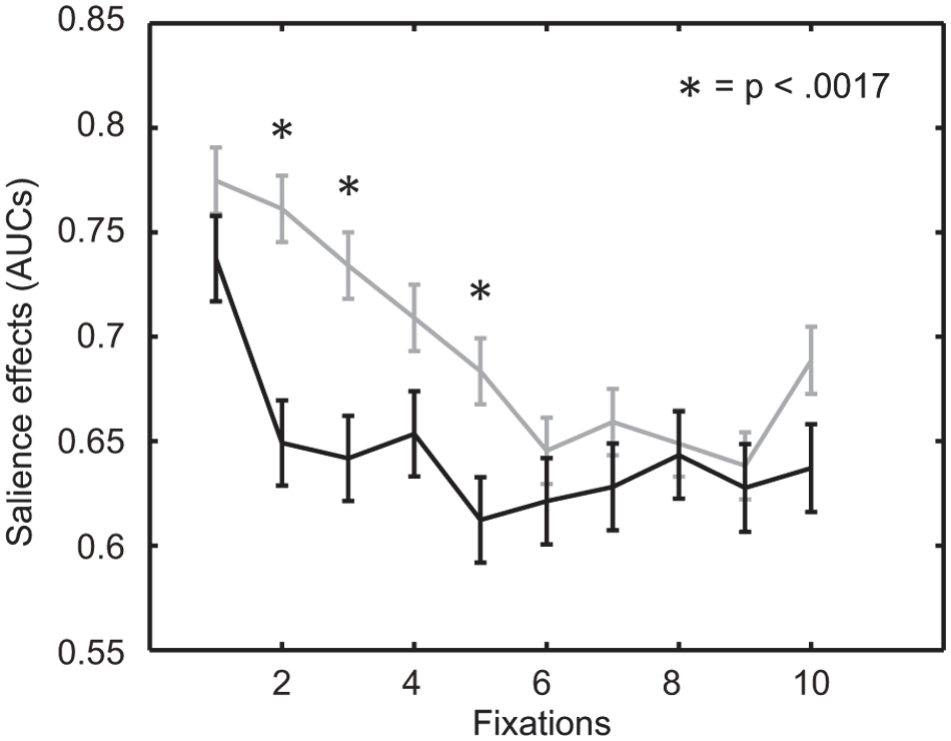
Variation of salience effects over fixations for the few-peaked group. Each of two polylines connects two means of salience effects for one fixation step and the next step. The black line represents salience effects for the artists. The gray line represents salience effects for the novices. The error bars represent standard error.

**Fig 6:**
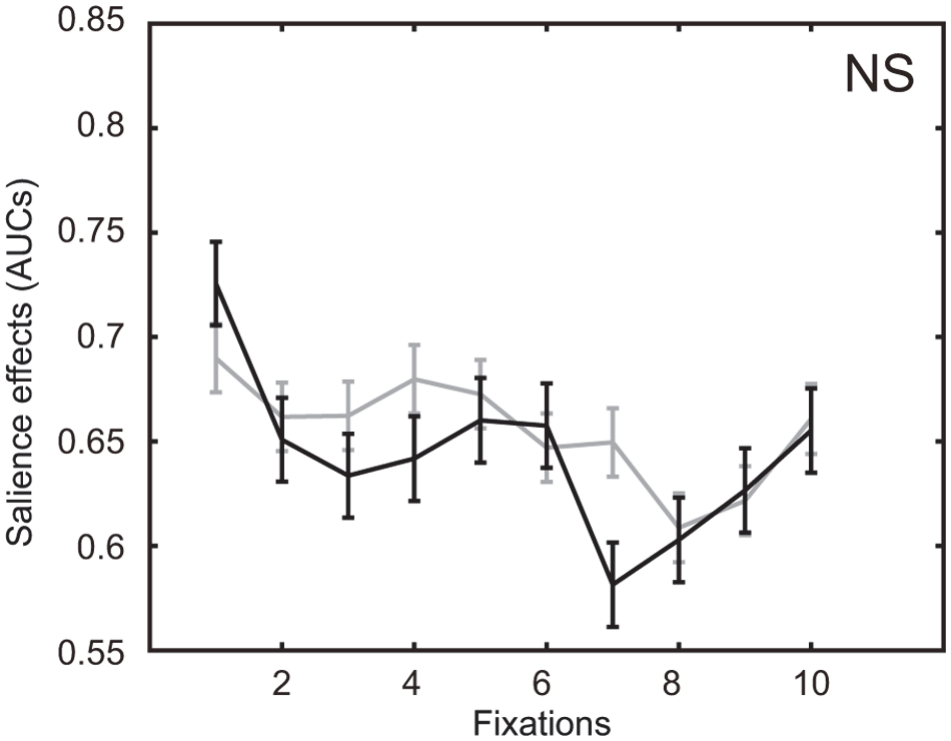
Variation of salience effects over fixations for the many-peaked group. Each of two polylines connects two means of salience effects for one fixation step and the next step. The black line represents salience effects for the artists. The gray line represents salience effects for the novices. The error bars represent standard error.

Multiple comparison tests (Bonferoni) comparing artists and novices were conducted for the salience effects of each of the bundles. Significant differences were observed in the second, third and fifth bundles in the few-peaked group of paintings, whereas the salience effects of the artists were lower than those of the novices (Fig. 5). In the many-peaked group of the paintings, a significant difference was not seen in each bundle (Fig. 6).

## Discussion

Our results suggest that the contribution of stimulus-driven factors in visual selection differed between artists and novices in the viewing of abstract paintings. In viewing abstract paintings, although Zangemeister *et al*. observed that art professionals show more global scanning than do novices [4], the authors did not describe attended features of artists compared with those of novices. On the other hand, in viewing representational images, artists’ fixations have been shown to be attracted less to the semantic objects than those of novices [6]. This implies that artists’ fixations are attracted more to non-semantic visual patterns than those of novices. Furthermore, there is an evidence of superiority of artists to recognize visual patterns [14, 15]. Therefore we expected that artists would have knowledge of non-semantic visual patterns, which led to a difference in contribution of stimulus-driven visual selection in viewing abstract paintings. Using a prediction model of stimulus-driven visual selection, we showed that artists’ fixations were less guided by local saliency than those of novices.

This difference in contribution of stimulus-driven visual selection was found only in paintings with few peaks of saliency. In viewing paintings of the few-peaked group, the artists’ salience effects tended to be lower than those of the novices. This result lends support to the idea that the clear saliency of peaks against the background may produce a pop-out effect [12]. For the novices, clear salient locations in paintings of the few-peaked group may enhance a stimulus-driven effect more than salient locations in the many-peaked group. In contrast, the artists’ fixations may be less guided by salient locations in viewing paintings of both the few-peaked and many-peaked groups, thereby resulting in the lower salience effects of the artists may be found only in the few-peaked group.

In our study, in free viewing of abstract paintings for five seconds, we found that the salience effects of both the artists and the novices were higher than those points randomly generated from a model distribution of fixations. Using the same duration of viewing paintings and the same model as our study used, Fuchs *et al*. also showed that salience effects in human fixations were higher than those of points generated from a center-bias distribution [17]. In another study, higher salience effects were found for human fixations over randomly generated points [12], although the quantification method of salience effect was not the same as our method. These results indicate that stimulus-driven factors contribute to fixation guidance for the first five seconds of free viewing. In our study, the contribution of stimulus-driven factors was also indicated in both the artists and the novices. The contribution, however, may be lower in the artists than in the novices because the salience effects were significantly lower in the artists.

Regarding the time sequence of salience effects, lower salience effects of the artists were found only in early fixations, typically excluding the first few fixations, where visual selection is modeled to be predominantly stimulus driven [11]. After the salient stimuli has captured the attention, top-down factors play a role in visual selection [32]. For the first few fixations, both artists and novices may be mainly influenced by a stimulus-driven factor. Then, for subsequent fixations, artists’ fixations may be less guided by stimulus-driven factors compared with novices. Tatler *et al*. suggest that the decrease in salience effects over time is caused by a lack of consideration of central bias of both the early fixations, and salient locations [23]. The decrease in salience effects over time for the artists was possibly due to their fixations moving away from the center faster than those of the novices. Therefore in the case of our study, as an additional analysis, we examined whether the distribution of saliency maps was equally center biased as distribution of actual human fixations or not. We calculated the distance between the random points generated from each saliency map and the center of a stimulus. On the other hand, the distance from the center was also calculated for random points selected from the distribution of actual fixations for the first five seconds, as center bias distribution. As a result, the distances for saliency maps for all paintings were significantly higher than those for the center-bias distribution, Welch’s test (*p* < .001). This indicates that the center-bias fixations could not ensure higher salience effects, and fixation bias does not cause lower salience effects in the artists. Lower salience effects in artists may be caused by their knowledge acting as a top-down factor. Considered a trade off between stimulus-driven and knowledge-based influences [33], the knowledge-based factor can be thought to contribute in guiding the artists’ fixations more than those of the novices.

For other domains, fixations of the specialist group were also less correlated to salient locations when viewing images related to their domain [20–22]. The authors suggest that domain-specific knowledge overrides stimulus-driven factors. In Humphrey’s study, visual memory task was imposed on participants to assure their expertise because specialists had shown superior performance in memory and cognitive tasks [20]. On the other hand, in our study, the task was that of a free viewing. Even so, expertise of the artists was also considered to be retained even in the free viewing task. In free viewing of representational images, artists have shown different visual selection from that of novices, in that they showed shorter fixation durations on the semantic objects [6], implying a difference in visual selection in the case of free viewing of images. Furthermore, another study also showed superior visual perception of artists in free viewing of paintings [7]. In this study, brain activity patterns of art professionals, compared with those of novices, showed higher accuracy for discrimination of art styles. We assess that expertise of artists was shown in free viewing of paintings in our experiments and this expertise, viewed as domain-specific knowledge, overrode stimulus-driven factors as suggested in [20–22].

Compared with expertise of other specialists such as Engineers and American study students in [20], artists’ expertise is more ambiguous, being less concrete and perhaps related to affection or other subjective factors. However, our results suggest that the knowledge of experts also overrides the stimulus-driven guidance of fixations. The results of our study imply that the artists attended more to visual patterns that were not described by merely salient features, compared with the novices. Considering their superior accuracy of pattern recognition [7, 14, 15], artists may have a richer knowledge of more complex visual patterns than salient features in recognizing the detailed visual differences. Indeed, in the V4 complex which is a higher visual system than regions related to salient features [10, 11], painting majors showed increased gray matter density and also increased correlation between the left V4 and the left prefrontal cortex, compared with those of novices [34]. Functional and structural differences in the V4 complex may result in painting majors having superior performance in discriminating subtle visual patterns. In that case, such superiority of artists reflects a higher contribution of knowledge-based guidance of fixations, when compared with novices.

In summary, to determine if the visual selection of artists differs from that of novices in the viewing of abstract paintings, we examined the contribution of stimulus-driven factors in visual selection. Our results showed that artists’ fixations were driven less by salient features than novices’ fixations. This indicates that artists’ knowledge overrides stimulus-driven factors guiding fixations during viewing of abstract paintings.
